# Talc-dominated seafloor deposits reveal a new class of hydrothermal system

**DOI:** 10.1038/ncomms10150

**Published:** 2015-12-22

**Authors:** Matthew R. S. Hodgkinson, Alexander P. Webber, Stephen Roberts, Rachel A. Mills, Douglas P. Connelly, Bramley J. Murton

**Affiliations:** 1 National Oceanography Centre, Waterfront Campus, Southampton SO14 3ZH, UK; 2 Ocean and Earth Science, National Oceanography Centre Southampton, University of Southampton, Southampton SO14 3ZH, UK

## Abstract

The Von Damm Vent Field (VDVF) is located on the flanks of the Mid-Cayman Spreading Centre, 13 km west of the axial rift, within a gabbro and peridotite basement. Unlike any other active vent field, hydrothermal precipitates at the VDVF comprise 85–90% by volume of the magnesium silicate mineral, talc. Hydrothermal fluids vent from a 3-m high, 1-m diameter chimney and other orifices at up to 215 °C with low metal concentrations, intermediate pH (5.8) and high concentrations (667 mmol kg^−1^) of chloride relative to seawater. Here we show that the VDVF vent fluid is generated by interaction of seawater with a mafic and ultramafic basement which precipitates talc on mixing with seawater. The heat flux at the VDVF is measured at 487±101 MW, comparable to the most powerful magma-driven hydrothermal systems known, and may represent a significant mode of off-axis oceanic crustal cooling not previously recognized or accounted for in global models.

## Abstract (web summary)

The Von Damm Vent Field comprises 85–90% talc, with hydrothermal fluids of unusual chemistry at 215 °C. Here, the authors show that the mineralogy results from seawater mixing with hydrothermal fluids and the heat output may represent a mode of crustal cooling not fully accounted for in global models.

## Graphical abstract

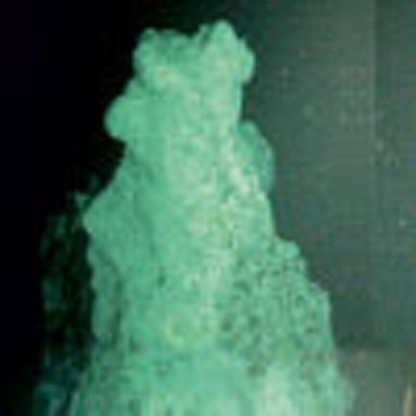

Hydrothermal activity at mid-ocean ridges is dominated by basalt-hosted, high-temperature, metal-rich vent systems driven by magmatic activity^1^. However, the discovery of ultramafic-hosted hydrothermal vent fields (for example, the high-temperature Rainbow and the low-temperature Lost City Vent Fields) demonstrates the diversity of hydrothermal activity associated with medium-slow spreading ridges^2^,^3^. Tectonic exposure of upper mantle and lower crustal rocks gives rise to a more heterogeneous basement than at intermediate-fast spreading ridges, and detachment faulting provides pathways to enhance hydrothermal circulation^4^,^5^.

A new class of hydrothermal system, the Von Damm Vent Field (VDVF), was discovered in the Caribbean during cruise JC044 of the RRS *James Cook* in April 2010, which hosts a community of hydrothermal vent fauna similar to those at the Mid-Atlantic Ridge^6^. The VDVF hydrothermal plume is rich in methane, has a significant Eh anomaly (indicative of reduced fluids) and is free of metallic particulates^6^. Here we describe how the unusual mineralogy of the VDVF results from mixing between moderate-temperature vent fluid and cold seawater. The processes leading to the formation of the VDVF may be widespread throughout medium to ultraslow mid-ocean spreading ridges medium-ultraslow spreading mid-ocean ridges and could play a significant role in the cooling and chemical exchange between oceanic crust and seawater.

## Results

### Geological setting

**Figure 1:**
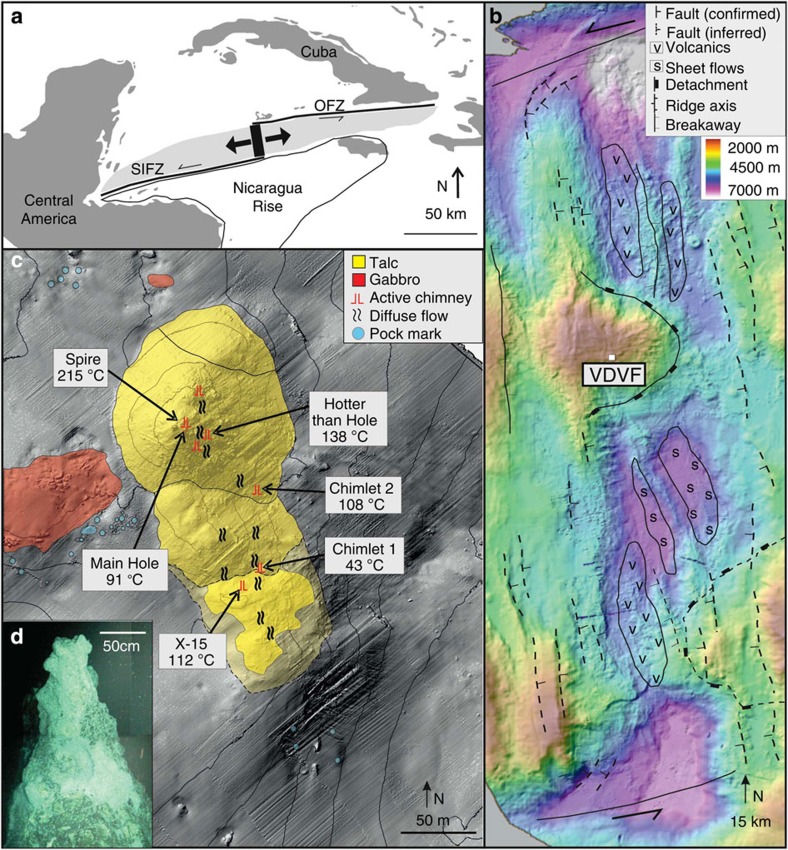
Location and bathymetry of the VDVF. (**a**) map of the Caribbean showing the location of the Mid-Cayman Rise and the black rectangle represents the area of **b**; the lightly shaded area is the area occupied of the Cayman Trough; OFZ, Oriente Fracture Zone; SIFZ, Swan Island Fracture Zone. (**b**) Bathymetry and interpretative geology of the Mid-Cayman Rise showing regional tectonic structures. (**c**) Bathymetry of the active VDVF showing the location of hydrothermal activity across the vent field. Contours are at 20-m intervals. (**d**) Photomosaic of The Spire at the top of the main VDVF cone obtained from high-definition video.

The VDVF is located on the western flanks of the ultraslow spreading Mid-Cayman Spreading Centre, the deepest spreading centre on Earth (Fig. 1a). It is situated at a depth of 2,280 mbsl, 13 km to the west of the rift axis^6^,^7^ on the upper slopes of Mt Dent, an oceanic core complex (OCC) formed by detachment faulting (Fig. 1b). At this location, the basement age is estimated to be between 1 and 2 Ma (based on distance from spreading centre and spreading rate) and comprises meta-gabbro, dolerite dykes and serpentinized peridotites that are partially covered by calcareous pelagic sediment^7^. The hydrothermally active area of the VDVF comprises three overlapping conical-shaped talc mounds, up to 75 m high and 150 m in diameter, aligned north-northwest–south-southeast (NNW-SSE) (Fig. 1c). The summit of the largest mound (Main Cone), which is the northern mound at the active site, hosts a 3-m tall, 1-m diameter chimney (The Spire, Fig. 1d). Hydrothermal fluid venting (215 °C) from The Spire has low concentrations of particles and a pH of 5.8 (determined at standard temperature and pressure (STP)). A 1-m diameter orifice (Main Hole) located at the base of The Spire vents fluids of up to 91 °C. Elsewhere across the VDVF, smaller orifices vent fluids of up to 138 °C (for example, Hotter than Hole, Chimlets 1 and Chimlets 2—Fig. 1c). Talc rubble at the base of the *Main Cone* onlaps the surrounding calcareous sediment—a relationship reversed at the base of the southern mound, indicating an increase in the age of the mounds towards the south (Fig. 1c). A further series of hydrothermally inactive, conical-shaped talc mounds is located 700 m to the south and east of the three active mounds. These include a 90-m high, sediment-covered cone (Mystic Mountain), which has twice the volume of the Main Cone. Samples from these extinct mounds are similar in composition, mineralogy and texture to the active mounds. With an estimated sedimentation rate of between 2 and 5 cm ka^−1^ (ref. 8), a thickness of 1 m or more of pelagic sediment covering most of Mystic Mountain indicates that its construction by hydrothermal activity ceased at least 20,000 years ago.

### Petrology

**Figure 2:**
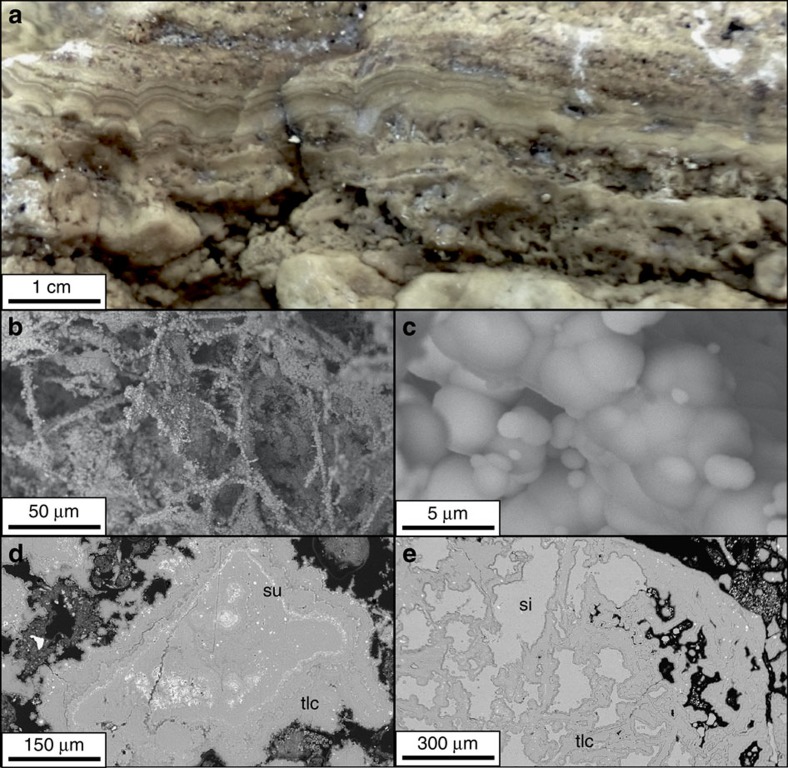
Hand-specimen and SEM images of VDVF samples. (**a**) Collapsed chimney wall showing bands of massive talc. (**b**) and (**c**) SEM images of dendritic and botryoidal talc networks. (**d**) SEM image of a cross section through botryoidal talc with bands of sulphide. (**e**) SEM image showing microcrystalline silica precipitated into a talc framework with disseminated sulphides. Legend: tlc=talc, su=sulphide, si=microcrystalline silica.

Compared with hydrothermally active seafloor deposits elsewhere, the mounds and chimneys of the VDVF are highly unusual, constituting 85–90% talc by volume with up to 10% microcrystalline silica and 5% disseminated sulphides (Supplementary Fig. 1). In hand-specimen, the hydrothermally active chimneys show millimetric layers of laminated and botryoidal talc (Fig. 2a). These layers are parallel and have an internal colloform structure (Fig. 2a). Under scanning electron microscope (SEM), broken surfaces reveal dendritic networks and botryoidal masses of talc, indicative of growth into open void spaces (Fig. 2b,c). Concentric bands of sulphide up to 50-μm thick are present as internal growth bands within the talc masses (Fig. 2d) and microcrystalline silica infills pore spaces (Fig. 2e). Talus forming the flanks of the mounds has a similar mineralogy to the venting chimneys, except that the botryoidal texture and open pore spaces are largely replaced by massive fine-grained talc, and the associated sulphides are mostly oxidized. Contrary to initial reports^6^, talc is the dominant mineralogy at the VDVF; only two of 50 samples recovered contain trace amounts of anhydrite and gypsum. Together with its botryoidal form, zoning, and layering. The talc deposits indicate precipitation from a hydrothermal fluid. The petrology of the hydrothermally active chimneys indicates a paragenetic sequence comprising initial talc growth into open space with co-precipitation of minor sulphide, followed by infilling of the pore space by later-stage microcrystalline silica. Loss of sulphides and a reduction in base-metal content in the flank talus indicate dissolution following exposure to cold and oxygenated seawater.

### Mineralogy

Bulk X-ray diffraction analyses (Supplementary Fig. 1) and microscopy of the VDVF chimney and mound material confirm the dominant presence of talc, with microcrystalline silica and sulphide constituting up to 15%. Chalcopyrite is the dominant sulphide (70%), indicative of episodically higher vent temperatures^9^, with the remainder being 20% pyrite, 5% sphalerite and 5% galena. The presence of sulphides at the VDVF, and a lack of sulphate in the end-member fluids, confirms a reducing environment for the precipitation of the chimney and mound talc deposits^10^.

Other studies of talc, recovered from seafloor environments elsewhere, have revealed significant amounts of intracrystalline layers of smectite clays reflecting the influence of sediment alteration and precipitation from Mg-rich pore waters^11^. X-ray diffraction analyses of unorientated air-dried mounts of talc separates from the VDVF chimneys and talus deposits show peaks at ∼9.6 Å (for the 001 plane) and ∼4.7 Å (Supplementary Fig. 2) confirming talc (Mg_3_Si_4_O_10_(OH)_2_) as the dominant phase. Shifts in peaks of the 001 plane from 9.604–9.627 Å for the air-dried mounts to 9.339–9.525 Å for the glycolated mounts show the presence of up to 10% of a smectite clay that is interlayered within the crystalline talc structure. The presence of peaks at 1.529 and 1.532 Å (for the 060 plane) indicate a tri-octahedral structure for the smectite clay interlayers (Supplementary Fig. 2). The dominance of talc (90%), with up to 10% clay interlayers, contrasts with previously reported, sediment-hosted seafloor talc deposits^11^.

### Deposit geochemistry

The high proportions of talc and microcrystalline silica are reflected in the whole-rock geochemistry (Table 1) by the high concentrations of SiO_2_ (52–66 wt.%) and MgO, (25–34 wt.%). Average base-metal concentrations in the hydrothermal chimneys are 1,463 p.p.m. Cu, 239 p.p.m. Zn and 112 p.p.m. Pb. In contrast, concentrations in samples of mound talus are an order of magnitude lower with averages of 241 p.p.m. Cu, 48 p.p.m. Zn and 15 p.p.m. Pb, consistent with the observed oxidation and loss by dissolution of sulphides in the talus samples.

**Figure 3:**
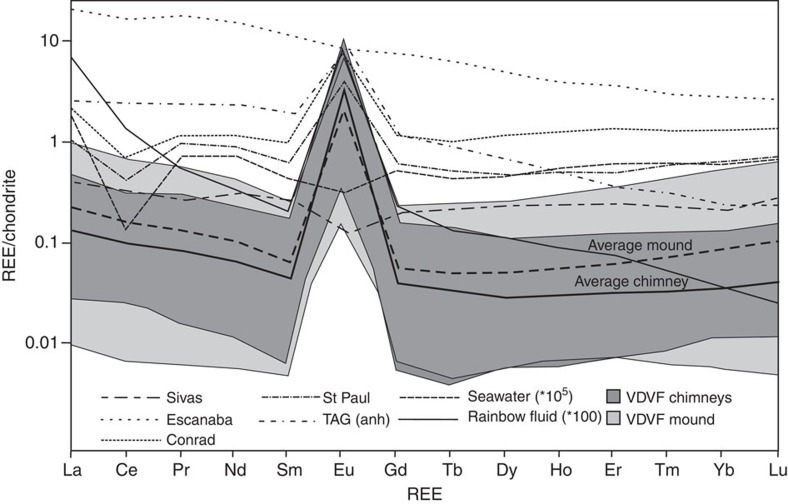
REE plots. Chondrite-normalized REE patterns showing the range and average concentrations of the VDVF mound (*n*=15) and chimney (*n*=9) materials. The patterns are characterized by light and heavy REE enrichment and large positive Eu/Eu* anomalies. Also shown for comparison are samples from the St Paul Fracture Zone, Conrad Fracture Zone, Escanaba Trough, (*n*=5), Sivas Basin (*n*=2), TAG anhydrite (*n*=24), Rainbow hydrothermal vent fluids (*n*=2) and seawater^2^,^17^,^54^,^55^,^56^.

Rare earth element (REE) patterns for talc, separated from hydrothermally active chimneys and mound talus samples, have shallow U-shaped profiles dominated by a large and positive europium anomaly (Fig. 3). Enrichment in the light RREs (LREEs) is indicated by La_(*N*)_/Sm_(*N*)_ (where *N*=chondrite-normalized values) ratios ranging between 0.9 and 10.4, with an average of 3.3 for chimneys and 3.1 for mound talus (Table 2). The REE profiles also have slight enrichment in heavy RRE (HREE) with average Dy_(*N*)_/Yb_(*N*)_ ratios of 0.8 for chimney material and 0.9 for the mound talus. The magnitude of the positive Eu anomaly, defined as Eu/Eu* (where Eu/Eu*=Eu_(*N*)_/√(Sm_(*N*)_ × Gd_(*N*)_), ranges between 6 and 228, with an average of 99 for the chimneys and 58 for the mounds (Fig. 3 and Table 2). The VDVF chimney and talus talc have ^87^Sr/^86^Sr ratios of between 0.706313 and 0.709168, respectively, which are similar to, but slightly less than, that of modern-day seawater (0.7092)^12^. This contrasts with the surrounding meta-gabbros that have ^87^Sr/^86^Sr ratios of between 0.702902 and 0.703657 (Tables 2 and 3).

The U-shaped REE patterns and positive Eu anomalies in the chimney and mound material indicate talc precipitation from VDVF vent fluid that has some similarities to high-temperature ‘black smoker' vent fluid REE chemistry^13^. Reducing conditions in the VDVF vent fluid, indicated by the presence of sulphides, enhance Eu mobility by the formation of divalent Eu chloride complexes, especially during the dissolution of plagioclase, resulting in large, positive Eu anomalies in fluids and precipitates^14^. Furthermore, the VDVF talc ^87^Sr/^86^Sr ratios indicate precipitation following mixing of the high-temperature VDVF vent fluid with a high proportion of seawater (of at least 10:1). Chlorinity in the end-member VDVF fluid of 667 mmol kg^−1^ is significantly elevated in comparison with seawater (546 mmol kg^−1^), enhancing the complexation of LREEs in relation to mid-RREs (MREEs) and HREEs in hydrothermal solutions at high temperatures and pressures, resulting in elevated LREE_talc_/MREE_talc_ ratios^15^. In contrast, moderate HREE enrichment is largely a crystallographic effect of the talc mineralogy, where HREEs substitute in the octahedral Mg site as a result of the ionic radii being of a more similar size compared with the LREEs^16^.

Other examples of seafloor talc deposits, reported from the St Paul and Conrad fracture zones, are inferred to have precipitated from the interaction of hydrothermal fluid with either seawater or a mafic protolith^17^. These talc samples have positive Eu anomalies and flat HREE profiles (Fig. 3), closely resembling those for the VDVF chimney and mound talus^17^. Seafloor talc deposits from elsewhere lack a positive Eu anomaly and have flat HREE profiles, consistent with formation as alteration products of an ultramafic protolith or sediment (Fig. 3)^17^,^18^. In contrast, the positive europium anomaly for the VDVF talc is consistent with primary precipitation from hydrothermal fluids. The radiogenic ^87^Sr/^86^Sr ratios for the talc further indicate that a significant component of seawater is mixed with the vent fluid during talc precipitation.

We conclude from the petrographic and geochemical evidence that the VDVF talc is a primary precipitate from a hydrothermal fluid mixed with seawater. To date, no other seafloor, talc-dominated, active hydrothermal vent field has been reported, making the discovery of the VDVF a new and unique class of hydrothermal system.

### Vent fluid chemistry

**Figure 4:**
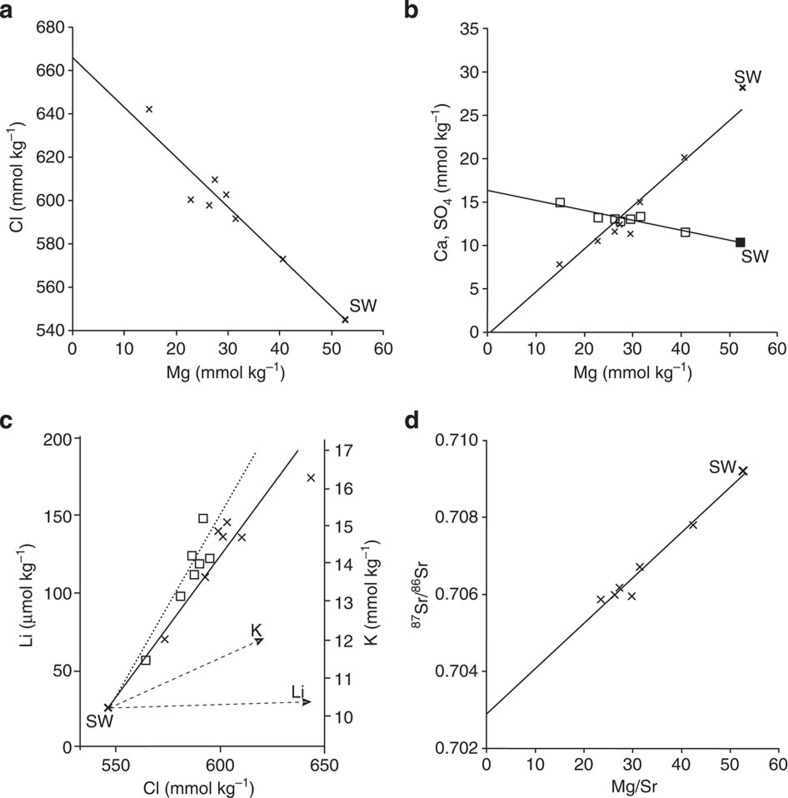
VDVF fluid plots. (**a**,**b**) The top diagrams show Cl, SO_4_ (crosses) and Ca (squares) with best fit lines extrapolated to zero Mg concentration. (**c**) Plots of Cl versus Li and K. The solid line and crosses indicate the linear trend of Li towards seawater (SW); the dashed line and squares indicate the linear trend of K towards SW. The arrows indicate the expected increase in Li, K and Cl concentration resulting from phase separation of seawater alone. (**d**) Ratios of ^87^Sr/^86^Sr plotted against Mg concentration in fluid samples and extrapolated to zero Mg/Sr to determine the VDVF end-member vent fluid ratio.

To explore whether the VDVF is currently precipitating talc, we sampled and analysed the composition of the vent fluids escaping from three different chimneys with a maximum measured temperature range of 108–215 °C (Table 3). It should be noted that the vent temperatures and fluid chemistry samples are decoupled, which precludes the possibility of extrapolating to an end-member temperature using fluid chemistry. Instead, we use the highest measured temperature of 226 °C (ref. 19) and assume that this approaches the end-member temperature. We find that our vent fluid samples lie on a mixing line between seawater and zero Mg (Fig. 4). This is consistent with an end-member vent fluid from which Mg has been quantitatively removed in the subsurface^20^. When extrapolated to zero Mg, the VDVF end-member fluid has a moderate pH of 5.8 (at STP; Table 3) and a dissolved Si concentration of 7.5 mmol kg^−1^ (ref. 21). End-member concentrations of K (17.5 mmol kg^−1^) and Li (241 μmol kg^−1^) are much higher than those generated by phase separation of seawater alone (Fig. 4c and Table 3), indicating significant exchange with subsurface host rocks^20^. Strontium isotopes for the VDVF fluid samples also lie on a mixing line against Mg/Sr with seawater indicating an end-member vent fluid ^87^Sr/^86^Sr of 0.702908, close to the basement rock values of 0.702902–0.703657 (Fig. 4d and Tables 2 and 3). Base-metal concentrations in the fluid samples range from 6.6 to 604 μmol kg^−1^ for Fe, 4.6–14.2 μmol kg^−1^ for Mn and 0.4–460.0 μmol kg^−1^ for Cu, but do not show conservative mixing with seawater and hence end-members cannot be derived (Table 3). Variation in base-metal concentrations and ratios between hydrothermal vents across the VDVF suggests subsurface processes of precipitation and/or zone refining within the talc mounds. Owing to highly variable concentrations, reliable end-member vent fluid Fe and Mn concentrations could not be determined. However, the range of concentrations of Fe and Mn are 10–1,000 times lower than those reported for the Rainbow ‘black smoker' end-member vent fluid^2^. In contrast, a chlorinity of 667 mmol kg^−1^ for the end-member fluid is 22% higher than ambient seawater (546 mmol kg^−1^), indicating a process of brine concentration.

The low metal concentrations as well as the near-neutral pH of the fluids venting at VDVF are consistent with temperatures in the water–rock reaction zone, which are significantly cooler than the ∼500 °C calculated for ‘black smoker' vents^22^. It is also known that elevated hydrogen sulphide concentrations in ‘black smoker' hydrothermal vents are related to magmatic input^23^. Hence, we suggest the low H_2_S concentrations at the VDVF are indicative of minimal magmatic contribution, consistent with the lower temperature of the end-member vent fluid and the ridge-flank setting of the vent field.

Increases in chlorinity for hydrothermal fluids interacting with an ultramafic basement have been suggested to occur in a number of ways: rock alteration, phase separation and brine and halite addition^2^,^24^. Temperatures in excess of 360 °C are required to phase-separate seawater at 200 bar (that is, at the depth of the VDVF) and even higher for deep subsurface reactions^25^. This is considerably hotter than the maximum temperature recorded at the VDVF^21^ and would require substantial subsurface cooling by seawater circulating deep within the talc mounds for which there is no evidence. The dissolution of residual halite, or the mixing with residual brine formed during an earlier and higher-temperature period of hydrothermal circulation and phase separation deep in the crust, could increase the end-member chlorinity^26^,^27^; however, the VDVF and surrounding area lacks any mineralogical evidence for a ‘black smoker'-like phase of venting. The process of serpentinization also has the potential to increase fluid chlorinity by the removal of water from the fluid^2^,^21^. Using the equation for the serpentinization of pure forsterite to serpentine and brucite (equation (1)), we calculate that the increase in chlorinity for the VDVF end-member fluid could result from serpentinization at a ratio of 944 g of forsterite to 1 kg seawater. The presence of any mineralogical Cl in serpentine makes this estimate a minimum.


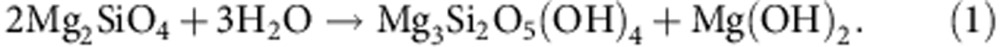


We have calculated a mass flux of end-member hydrothermal fluid at VDVF of ∼500 kg s^−1^ (see below) for which the increase in chlorinity requires brine expulsion following serpentinization of pure forsterite at a rate of at least 470 kg s^−1^. Alternatively, the entrainment of an early-formed brine or halite phase is consistent with a cooling hydrothermal system, while serpentinization is compatible with the tectonic setting of the VDVF on slowly exhumed lower-crust and upper-mantle rocks. Both processes are ultimately constrained by the availability of residual brine, halite or fresh peridotite.

### Fluid pH

A measured pH of 5.8 (at STP) for the VDVF end-member fluid is significantly higher than the observed range (pH 2.5–3.5) for sulphide-rich, high-temperature ‘black smoker' vent fluids. It is also lower than the alkaline, low-temperature fluids vented at the ultramafic-hosted Lost City Vent Field^3^,^20^. Hydrothermal fluids with intermediate pH (5–6) can result from the interaction of seawater with mafic and/or ultramafic lithologies at temperatures <300 °C (refs 28, 29). Under these conditions, pH is controlled by the balance between Mg removal from seawater and silicate hydrolysis (equation (2)). At low water/rock ratios (<10), the rate of magnesium consumption is relatively low, and silicate hydrolysis acts as a pH buffer (equation (3)). At high water/rock ratios (>50), H^+^ is produced at a rate greater than it is used up in hydrolysis reactions, resulting in lower pH^28^.


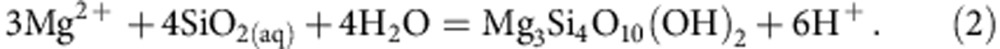



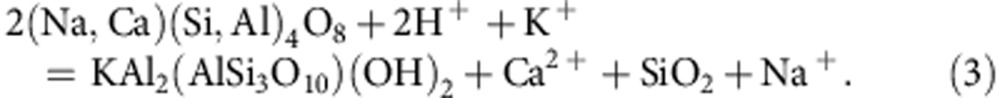


With its intermediate pH, 215 °C temperature and setting on lower-crustal and upper-mantle rocks, the fluids venting at the VDVF are consistent with moderate-temperature interaction within a gabbro/peridotite basement (equation (3)).

### Talc precipitation

**Figure 5:**
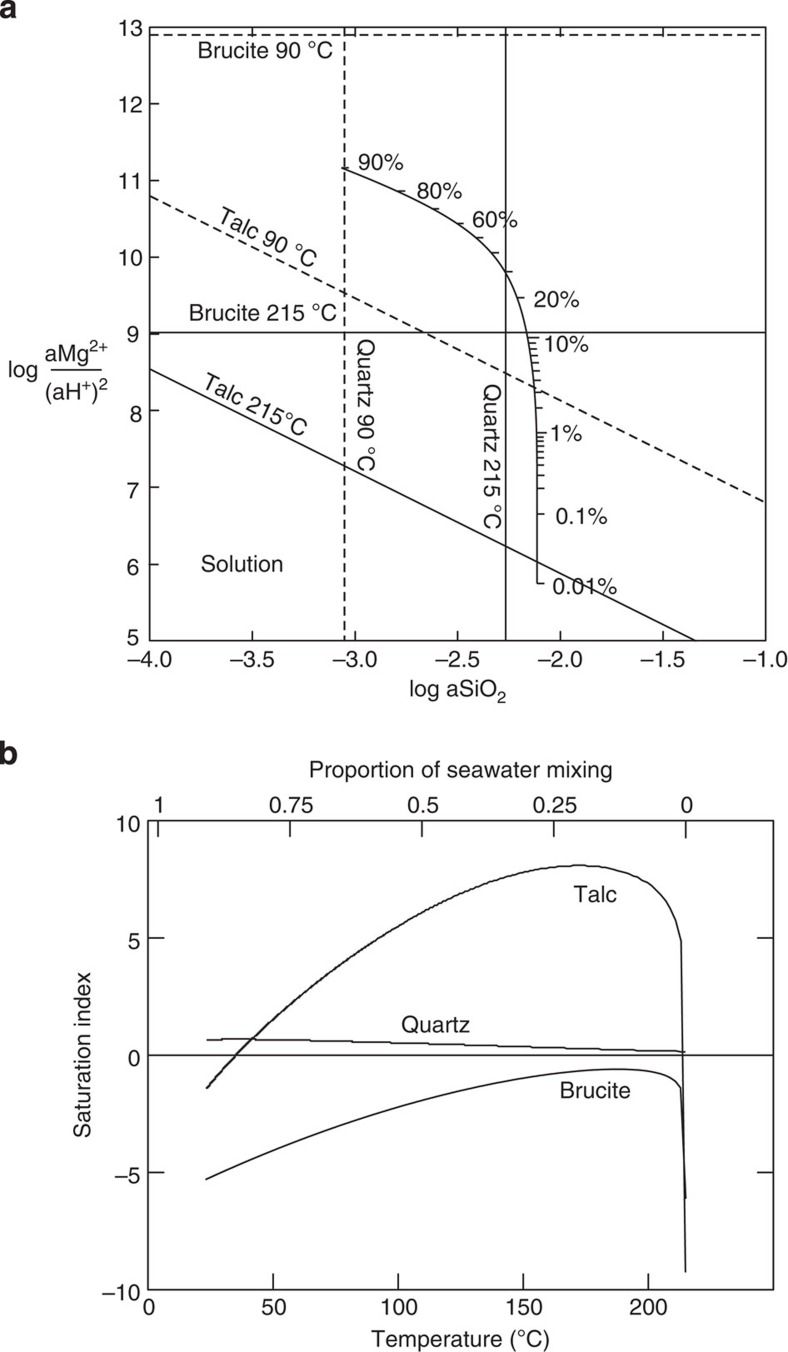
Thermodynamic phase diagram. (**a**) Stability boundaries of talc, quartz and brucite at 215 and 90 °C, calculated in Geochemist's Workbench (GWB) at the ambient pressure conditions of the seafloor at the VDVF. The percentages indicate the proportion of seawater mixing with the end-member VDVF fluid. (**b**) Saturation indices for talc, quartz and brucite during modelled mixing of VDVF end-member fluid with seawater from 0.01 to 99%. This indicates that talc becomes saturated on mixing end-member VDVF vent fluid with seawater, and remains stable throughout the mixing regime. Brucite, which is the dominant magnesium phase at the higher pH Lost City vent field, never reaches stability at the VDVF.

Thermodynamic modelling using the Geochemist's Workbench and SUPCRT92 (refs 30, 31, 32), under the ambient pressure and temperature conditions of the VDVF, predicts instantaneous precipitation of talc and silica as the primary phases on mixing the 215 °C VDVF end-member vent fluid with cold seawater (Fig. 5a). Both phases remain supersaturated throughout the mixing regime until seawater makes up ∼90%, and the fluid reaches ∼25 °C (Fig. 5b). Below this temperature, talc remains undersaturated that, together with the kinetics of the reaction, may explain why talc was not visibly precipitating in the hydrothermal plume. Oscillatory zoning of talc and silica within active chimneys indicates a fluid composition at the VDVF that fluctuates around the intersection of the talc–silica saturation limits in the H_2_O-HCl-(Al_2_O_3_)-MgO-SiO_2_ system. A fluid composition around the eutectic between talc and amorphous silica would be influenced by slight changes in Mg or Si activity, brought about either by precipitation of one or the other phase, or by fluctuating seawater proportions within the mound. Silica is saturated in the end-member fluid, therefore would precipitate when no seawater is available to provide Mg for talc precipitation. Such dynamic conditions, which are well documented at other sites^33^,^34^,^35^, could lead to alternating layers of mineral phases. Bands of chalcopyrite also indicate episodically higher temperatures. The prediction that talc precipitates directly as a result of mixing between the VDVF vent fluid and seawater is further supported by the range of ^87^Sr/^86^Sr ratios for the talc (0.706313–0.709168), which lie between the value of modern seawater (0.7092) and the VDVF end-member hydrothermal fluid (0.702908; Tables 2 and 3 and Fig. 4).

The dissolved silica concentrations in the VDVF end-member fluids are similar to those reported from ‘black smoker' vents^20^, where talc is also theoretically stable on mixing with cold seawater. However, the large quantity of sulphides precipitated at these vent sites results in talc and other silicates only occurring as accessory minerals (for example, Middle Valley)^36^. At the VDVF, the low metal content of the fluid results in only accessory amounts of metal sulphides, allowing talc and silica to become the dominant phases. By comparison, the higher pH of the Lost City vent fluids results in calcium carbonate and brucite precipitation instead of talc^3^.

In summary, the geochemistry of the solid and fluid phases and thermodynamic modelling are consistent with the observed texture of the talc, its growth into pore spaces, the presence of actively venting talc chimneys and the precipitation of significant volumes of talc-forming large conical mounds on the seafloor. We suggest that similar conditions prevailed during the formation of the other, now hydrothermally extinct, talc mounds (for example, Mystic Mountain) located ∼700 m to the east of the currently active site. The current VDVF represents the latest stage in talc precipitation and hydrothermal circulation in the mafic and ultramafic basements beneath the Mt Dent OCC over tens of thousands of years.

## Discussion

Hydrothermal venting on ocean crust more than 1 Ma in age has only been observed previously as either low-temperature (<10 °C) diffuse flow of reduced fluids^37^ or as medium-temperature (91 °C) venting of high pH fluids (for example, at the Lost City hydrothermal field^3^,^38^) forming brucite and carbonate chimneys. In contrast, the VDVF vents fluid in excess of 200 °C, from seafloor mounds and chimneys of predominantly talc, developed on a 1–2-Ma basement of lower-crust and upper-mantle rocks that were tectonically exhumed. This discovery, of an active hydrothermal system depositing predominantly talc as chimneys and forming large mounds on the seafloor, is unique.

While talc-rich deposits of postulated hydrothermal origin have been reported before^11^, these were mainly precipitated as accessory phases associated with massive sulphides, either where Si-rich low-pH hydrothermal fluids mixed with seawater, or as a result of conductive heating of Mg-rich sedimentary pore waters. Only two seafloor talc deposits, from the St Paul's and Conrad fracture zones in the Atlantic Ocean^17^, appear directly comparable to the VDVF in terms of mineralogy, texture and geological setting.

At both sites, botryoidal talc, with similar REE patterns to the VDVF talc, was recovered from a tectonically exhumed gabbro–peridotite basement^17^. The presence of similar material to the active VDVF deposits at two different sites suggests that talc-dominated seafloor hydrothermal mineralization may be a widespread process at other slow spreading ridges, where tectonic uplift exposes lower-crustal and upper-mantle rocks to alteration by moderate-temperature hydrothermal circulation.

By measuring vertical velocities of vent fluid as it exits the seafloor, *in situ* fluid temperatures and the diameters of vent orifices at the VDVF, we have calculated a focused hydrothermal heat flux of 487±101 MW, produced from the venting of ∼500 kg s^−1^ of end-member fluid (Table 4). Despite being dissipated from crust of 1–2 Ma, this heat flux is comparable to that reported for focused heat flux dissipated by magma-driven ‘black smoker' vent fields on zero-age ridge axes such as the Endeavour Ridge (302 MW), the East Pacific Rise at 21°N (200 MW) and the *TAG* vent field (758 MW) on the Mid-Atlantic Ridge^39^. When compared with other ridge-flank hydrothermal systems^37^, the heat flux at the VDVF is exceptionally high. It is also greater than the estimate for the Lost City hydrothermal field, which is thought to derive heat from the exothermic reaction of serpentinization^40^,^41^ in addition to residual crustal heat^42^.

While hydrothermal activity is effective at extracting heat from the magmatic ridge axis^43^,^44^, processes of residual heat extraction from ridge flanks remain unclear. The intermediate temperature, near-neutral pH, low H_2_S and low concentration of dissolved metals in the VDVF vent fluid are all features consistent with hydrothermal circulation driven by residual heat within the tectonically uplifted lower crust and upper mantle^45^. Despite a high heat flux, the neutrally buoyant VDVF hydrothermal plume contains very little particulate matter^6^ and, as a result, is difficult to detect using conventional optical sensors. Given the widespread occurrence of OCC's exhuming lower-crustal and upper-mantle rocks at ultraslow-medium spreading ridges, and the presence of similar talc deposits at the St Paul's and Conrad fracture zones^17^, we suggest that the processes leading to the formation of the VDVF could be widespread at slow-ultraslow spreading. In that case, the VDVF class of hydrothermal activity could contribute significantly to the cooling of the oceanic crust, but are not yet included in current estimates of global hydrothermal activity based on plume incidence^46^.

## Methods

### Sample collection

Sonar data, photographic and video imagery and samples of rocks, minerals, fluids and temperatures were acquired using remotely operated vehicles (*Isis* and *HyBIS*) during RRS *James Cook* cruises JC44 and JC82.

### Vent fluids

High-temperature fluids were sampled using titanium gas-tight syringes at three different high-temperature chimneys (Table 3). Fluid exit temperatures were determined separately from the fluid sampling using a laboratory-calibrated, high-temperature thermal probe that was inserted deep into each vent orifice. Hence, the fluid samples and temperatures are decoupled. The highest measured value for each vent is reported in Table 3. Subsamples of vent fluids were taken for geochemical analyses, with pH, halides, sulphate and H_2_S being determined at STP (25 °C) immediately after recovery of the titanium syringes on the surface. Anions were determined using ion chromatography, and cations using inductively coupled plasma atomic emission spectroscopy and inductively coupled plasma mass spectrometry (ICP-MS) at NOCS. Data quality was assessed using the ‘Mottl Vent Fluid Database' protocols^47^. This included analysis of all solid precipitates from within the sampling syringes following total dissolution and weighted volumetric addition of concentrations to the clear fluid samples. Hence, any precipitates after the fluid samples were taken were re-dissolved and accounted for in final concentrations. Quality control included screening for samples with excessive Mg on a regression of elements such as Si, Ca, K and Li to zero Mg. Samples that were found to be contaminated with Mg concentrations more than those of seawater were subsequently excluded from the database. Our end-member concentration for dissolved silica is not as well constrained as that obtained in ref. 21, and thus their concentration is cited and used in our thermodynamic modelling.

### Petrography

Petrographic analyses were conducted using transmitted and reflected light microscopy on polished thin sections. SEM was conducted using two different systems: one using a low-resolution Hitachi TM1000 Desktop SEM operating at 15 kV, imaging 1-cm^2^ blocks of untreated broken surfaces; the second was a high-resolution LEO 1450-VP SEM operating at 20 kV and imaging carbon-coated polished thin sections. Crystallographic analysis was made using X-ray diffraction with Cu Kα radiation 2*θ* ranging between 2° and 76°, at steps of 0.02° and at rates of 0.02° per s. Unorientated air-dried mounts and glycolated mounts were scanned in the ranges 0–20° and 0–40°. Randomly orientated mounts were prepared and scanned in the range 57–62° to identify the peak signifying the (060) crystallographic plane.

### Geochemistry

X-ray fluorescence (XRF) analysis was conducted on fused beads in a 10:1 ratio of sample powder to lithium tetraborate flux. Precision was determined from repeat analysis of standards UB-N, BRR-1 and OPY-1, and gives errors of <10%. Talc mineral separates were handpicked and washed in a weak acid solution and rinsed in de-ionized water. Following hydrofluoric and nitric acid digestion, the samples were analysed for trace element and REE analyses using ICP-MS. Standards included international reference materials: BIR-1, JB1a, JGB1, JB-3 and BHVO-2. Precision was determined from repeat analysis and gives errors in the range of 0.21–5.71%. Whole-rock analysis was conducted using both ICP-MS and inductively couple plasma atomic emission spectroscopy, and calibration was made using synthetic multielement standards. ^87^Sr/^86^Sr ratios were obtained using a thermal ionization mass spectrometer. Fluid and host rock samples were analysed by running through Sr-spec^Tm^ resin to obtain 1 μg of strontium. Samples of strontium (200 μg) were obtained from pure talc by running through cation resin AG50-X8 200–400 and then through Sr-spec^Tm^ resin. External reproducibility of analysis was checked by analysing standard NBS987 every 11 samples and is reported here with an average of 0.710250 (1*σ*=0.0000053), which is within an error of the accepted value of 0.710254.

### Thermodynamic modelling

Modelling was carried out using Geochemist's Workbench^30^ at pressure and temperature conditions appropriate for the depth of the VDVF. A thermodynamic database for the composition of the vent fluid and seawater, at 250 bar and 4–215 °C, with logK values calculated through this range of temperatures, was generated using DBCreate^31^, which in turn uses SUPCRT92 (ref. 32). Thermodynamic properties for aqueous species and phases were taken from the 2006 revision of the SUPCRT database^32^.

### Heat flux calculation

Focused hydrothermal flow rates were calculated from high-definition video footage of the vertical velocity of particles entrained within venting hydrothermal fluid.

Individual particles entrained in the exiting vent fluid were filmed over a rise height of ∼1 m and their velocity calculated from frame to frame. The distance travelled by the particles was measured against two parallel laser beams separated by a constant distance of 10 cm and shone through the rising vent fluid. The fluid exit velocities at each vent orifice were determined by measuring multiple particles in the upflowing fluid across the entire dimensions of the vent orifices. Different particles entrained in the up-flow zone from each orifice displayed only slight variation in their velocities, indicating that the flow rates are relatively uniform for fluids exiting each vent orifice (Supplementary Table 1). The velocity of a number of particles was thus calculated over a period of several minutes and a mean velocity derived for vent fluid as it escaped the vent orifice. The calculated error in the velocities is derived from the variation in particle velocity. These data were then integrated for each orifice as per established methods^48^,^49^,^50^,^51^,^52^.

This method models the vent orifices as circles, and makes the assumptions that the fluid is emanating from the vent at a constant velocity across its diameter, the temperature is uniform across the vent orifice and the particle velocities recorded were representative of the flow velocity. Specific heat capacity of the venting fluids was calculated using specific densities and thermal capacities for seawater^53^ at the temperature and pressure conditions recorded at the VDVF.

## Additional information

**How to cite this article:** Hodgkinson, M. R. S. *et al*. Talc-dominated seafloor deposits reveal a new class of hydrothermal system. *Nat. Commun.* 6:10150 doi: 10.1038/ncomms10150 (2015).

## Supplementary Material

Supplementary Information Supplementary Figures 1-2 and Supplementary Table 1

## Figures and Tables

**Table 1:** Whole-rock geochemistry.

Type	Sample	SiO_2_(wt.%)	TiO_2_(wt.%)	Al_2_O_3_(wt.%)	Fe_2_O_3_(wt.%)	MgO (wt.%)	CaO (wt.%)	K_2_O (wt.%)	Na_2_O (wt.%)	P_2_O_5_(wt.%)	LOI (wt.%)	Sum (wt.%)
Mound	198-03	62.12	0.01	0.27	0.16	31.26	0.08	0.04	0.18	0.01	5.70	99.81
Mound	198-04	56.54	0.01	2.75	1.15	30.08	0.20	0.09	1.27	0.00	7.66	99.75
Mound	198-05	57.88	0.02	0.70	0.68	29.51	1.81	0.10	0.43	0.02	8.50	99.65
Mound	199-09	60.90	0.01	0.46	0.28	31.18	0.19	0.05	0.22	0.01	7.20	100.50
Mound	199-10	66.11	0.01	0.18	0.27	28.10	0.08	0.03	0.13	0.00	5.50	100.41
Mound	200-21	63.66	0.01	0.18	0.39	28.90	0.10	0.05	0.12	0.01	8.55	101.96
Mound	200-23	59.91	0.01	0.17	0.28	31.41	0.27	0.09	0.22	0.02	7.30	99.68
Chimney	200-24	57.61	0.01	0.02	0.81	28.33	0.41	0.14	0.68	0.02	11.60	99.63
Mound	201-34	61.12	0.01	0.04	0.44	32.10	0.22	0.12	0.52	0.02	5.70	100.27
Mound	201-36	58.51	0.01	1.00	0.71	30.53	0.15	0.07	0.44	0.01	7.70	99.13
Chimney	201-37	53.51	0.01	0.24	0.57	30.71	0.06	0.07	0.24	0.02	9.63	95.04
Mound	201-38	58.39	0.01	0.31	0.29	31.77	0.37	0.09	0.38	0.01	9.10	100.70
Mound	201-39	54.26	0.09	2.04	0.90	34.19	0.06	0.02	0.22	0.12	8.79	100.68
Mound	201-40	56.47	0.01	0.60	0.38	32.70	0.12	0.07	0.37	0.01	8.77	99.50
Chimney	202-43	57.99	0.01	0.19	0.33	31.81	0.18	0.10	0.43	0.01	8.30	99.33
Chimney	202-45	51.85	0.01	0.05	0.41	31.04	0.32	0.07	0.35	0.06	15.10	99.25
Chimney	202-46	60.05	0.01	0.07	0.34	30.58	0.18	0.06	0.25	0.02	12.17	103.73
Chimney	202-48	66.22	0.01	0.71	0.37	25.51	0.15	0.06	0.43	0.01	6.26	99.71
Chimney	202-49	54.83	0.01	0.05	0.49	30.79	0.32	0.07	0.31	0.03	12.76	99.65
Chimney	199-107	58.31	0.01	0.65	0.64	30.92	0.15	0.07	0.40	0.01	8.78	99.94
Chimney	199-110	57.05	0.01	0.19	0.19	30.73	0.21	0.08	0.41	0.02	11.02	99.91
Chimney	44-1E	58.12	0.01	0.44	1.13	27.81	0.12	0.09	0.59	0.01	11.50	99.80
Mound	44-2G	58.50	0.02	0.58	0.52	30.34	1.01	0.10	0.52	0.04	9.03	100.66

As (p.p.m.)	Ba (p.p.m.)	Cd (p.p.b.)	Cs (p.p.b.)	Cu (p.p.m.)	Mn (p.p.m.)	Nb (p.p.b.)	Ni (p.p.m.)	Pb (p.p.m.)	Rb (p.p.b.)	Sb (p.p.b.)	Sr (p.p.m.)	Zn (p.p.m.)
2.3	6.0	198	21	51.7	124.3	14	3.1	12.8	434	292	2.9	26.9
1.0	7.3	26	28	44.8	107.6	16	1.2	7.4	710	44	16.8	14.4
3.2	12.0	108	344	506.5	163.9	269	7.2	22.8	2,148	260	47.9	72.7
1.0	0.3	14	40	82.5	151.2	22	2.6	1.2	636	44	8.9	12.3
0.9	0.1	228	15	154.8	133.4	4	1.2	35.4	270	325	2.4	47.3
5.4	1.3	316	60	1,292.7	182.1	4	1.5	66.1	569	1,696	3.4	161.8
2.3	143.6	25	111	100.2	143.5	107	6.0	2.2	1,589	87	26.2	19.9
0.9	0.6	45	111	4.2	2,721.0	4	0.6	0.2	1,826	43	28.2	2.6
2.8	1.0	34	90	31.7	1,518.5	14	3.7	2.7	1,500	136	13.0	8.7
1.3	0.8	16	39	110.0	239.3	20	0.6	12.6	771	40	11.6	44.5
2.4	0.4	3,249	71	4,307.7	233.6	12	3.1	324.5	794	2,402	10.4	569.4
2.5	0.7	63	80	250.9	279.3	46	8.8	21.3	1,305	328	25.7	62.9
1.3	1.2	13	64	117.1	148.9	76	6.5	2.2	1,045	107	9.3	29.7
4.7	0.2	22	49	253.0	165.3	11	5.7	19.8	1,047	454	14.5	43.9
8.1	0.8	2,028	107	970.2	463.9	23	4.7	116.6	1,216	3,767	11.0	234.7
43.3	0.8	2,807	47	1,165.3	328.4	23	14.0	5.3	820	8,229	171.9	52.9
75.3	0.8	2,791	51	1,663.4	237.1	16	5.7	25.7	767	25,719	68.0	123.3
5.8	0.3	848	35	471.1	175.2	4	1.6	20.2	495	4,703	7.2	77.9
65.8	1.7	2,431	47	1,948.8	277.6	4	6.5	31.6	839	20,425	154.3	144.2
2.6	2.0	134	63	1,178.4	205.0	84	2.7	23.1	819	442	9.8	43.3
2.4	0.9	120	66	1,416.4	240.5	109	2.6	16.8	1,154	257	14.1	33.1
20.7	0.3	5,975	83	5,871.7	212.9	12	9.7	658.9	873	25,042	8.4	1,272.7
5.4	5.2	63	134	588.9	551.5	372	13.1	70.5	1,938	798	59.1	148.2

ICP-AES, inductively couple plasma atomic emission spectroscopy; ICP-MS, inductively coupled plasma mass spectrometry; VDVF, Von Damm Vent Field; XRF, X-ray fluorescence.

Major and trace elements within hydrothermal active samples from the VDVF. Major elements were derived by XRF and trace elements derived by ICP-AES and ICP-MS.

**Table 2:** REE geochemistry.

Type	Sample number	La (p.p.b.)	Ce (p.p.b.)	Pr (p.p.b.)	Nd (p.p.b.)	Sm (p.p.b.)	Eu (p.p.b.)	Gd (p.p.b.)	Tb (p.p.b.)	Dy (p.p.b.)	Ho (p.p.b.)	Er (p.p.b.)	Em (p.p.b.)	Yb (p.p.b.)	Lu (p.p.b.)	^87^Sr/^86^Sr
Mound	198-03	46.8	157.8	7.0	20.3	2.8	126.0	2.8	0.3	1.8	0.4	1.3	0.2	1.0	0.2	0.708685
Mound	198-04	4.0	9.8	1.4	6.5	2.3	16.6	4.5	0.8	5.7	1.3	3.9	0.6	4.3	0.6	0.708937
Mound	198-05	243.1	430.5	55.5	204.1	39.0	74.9	36.0	5.7	37.1	8.4	26.0	4.3	31.0	5.2	N.D.
Mound	199-09	36.8	83.4	8.7	34.6	7.4	83.4	9.7	1.5	10.7	2.5	8.0	1.3	9.5	1.7	N.D.
Chimney	199-10	19.7	68.1	4.8	17.8	2.6	109.6	3.0	0.4	2.4	0.7	2.3	0.3	2.3	0.3	N.D.
Chimney	199-107	19.8	49.5	6.1	25.5	6.6	77.7	9.2	1.2	7.7	1.6	4.3	0.6	4.2	0.7	N.D.
Chimney	199-108	7.3	17.6	2.0	9.0	2.4	20.9	3.4	0.5	3.6	0.8	2.6	0.4	2.8	0.5	0.709032
Chimney	199-110	12.1	28.2	3.6	15.0	3.5	58.2	5.0	0.8	5.3	1.3	4.1	0.7	6.2	1.2	0.709067
Mound	200-23	94.2	176.2	21.7	81.1	17.2	143.8	17.7	3.0	20.8	5.0	16.8	2.9	22.7	3.8	0.707526
Chimney	200-24	35.2	72.0	8.3	33.7	6.6	176.0	6.6	0.7	4.0	0.9	2.3	0.3	2.0	0.3	0.708375
Mound	201-34	63.7	173.6	17.9	68.5	9.7	372.5	10.7	1.2	7.6	1.7	5.5	0.8	5.3	0.8	N.D.
Mound	201-35	7.7	26.7	3.3	16.4	5.3	21.2	6.9	1.1	6.4	1.3	3.9	0.6	5.0	0.8	0.708949
Mound	201-36	23.5	52.1	5.6	22.1	4.5	75.2	5.1	0.6	3.3	0.7	1.5	0.2	1.1	0.2	0.708770
Mound	201-38	11.4	28.3	3.4	15.9	5.0	77.7	8.6	1.2	7.8	1.7	4.5	0.6	3.9	0.7	0.709083
Mound	201-39	12.5	25.5	3.0	12.2	1.9	102.9	2.3	0.2	1.9	0.4	1.3	0.2	1.3	0.2	N.D.
Mound	201-40a	5.9	11.0	1.7	7.2	1.7	94.8	3.3	0.5	4.0	1.1	3.7	0.5	3.6	0.5	0.709168
Mound	201-40b	2.6	4.5	0.6	2.9	0.8	38.9	1.4	0.2	1.5	0.4	1.3	0.2	1.0	0.1	N.D.
Chimney	202-43	21.0	30.3	4.3	16.8	3.4	275.3	4.4	0.6	4.6	1.2	3.6	0.6	3.3	0.6	0.708824
Chimney	202-47	8.5	19.5	1.7	6.1	1.0	84.2	1.2	0.2	1.7	0.4	1.3	0.2	2.2	0.4	0.708308
Mound	202-48	3.7	11.0	1.4	6.7	2.0	9.6	3.3	0.5	3.8	0.9	2.9	0.5	3.2	0.6	0.706313
Chimney	44-1A	114.9	193.9	28.8	109.3	26.4	617.4	32.2	5.3	27.8	6.7	20.7	3.2	21.6	4.0	N.D.
Chimney	44-1F	48.7	85.6	12.3	47.4	8.2	260.0	9.8	1.7	9.7	2.2	7.3	1.4	9.1	1.4	N.D.
Mound	44-2C	73.4	153.5	19.3	76.3	15.9	119.7	15.7	2.5	18.5	4.7	16.5	3.6	36.3	7.7	N.D.
Mound	44-2O	164.0	179.3	39.8	158.3	31.9	404.1	47.7	9.2	65.2	17.1	57.7	11.2	88.7	16.3	N.D.
Host	199-11	1,149.7	3,549.6	535.8	2,545.0	762.8	387.0	948.8	167.4	1,116.2	238.1	710.7	111.6	773.9	119.1	0.702902
Host	199-14	1,702.2	6,215.1	1,121.0	6,194.4	2,104.9	826.2	2,748.4	485.8	3,155.3	660.1	1,893.2	282.3	1,822.6	265.7	0.703189
Host	199-18	6,570.5	30,208.5	2,562.0	12,293.1	3,551.1	1,212.2	4,294.8	736.2	4,569.9	948.2	2,621.5	383.0	2,394.7	345.8	0.703657
Host	199-19	1,951.4	7,873.6	821.3	4,272.1	1,495.6	742.1	2,087.1	365.4	2,358.3	501.0	1,378.8	199.7	1,262.0	184.6	0.702997

N.D., not determined; REE, rare earth element; VDVF, Von Damm Vent Field.

REE concentrations and ^87^Sr/^86^Sr in pure talc and host rock from the hydrothermally active VDVF. Samples were all recovered during cruises JC044 and JC082 (refs 57, 58). Please refer to the Methods section for analytical precision and errors.

**Table 3:** Fluid data.

Sample number		198-GT1	198-GT2	200-GT1	200-GT3	200-GT4	202-GT3	202-GT4	End-members			Seawater
Site		The Spire	The Spire	Hotter than Hole	Hotter than Hole	Hotter than Hole	Chimlet 2	Chimlet 2	VDVF	Rainbow	Lost City	
Depth	m	2,291	2,291	2,307	2,308	2,308	2,379	2,379	2,379–2,291	2,300	700–800	
Max temperature	°C	215	215	138	138	138	108	108	215	365	91	4
pH		6	6.2	6.2	6.1	6.2	6.2	7	5.8	2.7	9	8.2
Cl	mmol kg^−1^	643	592	610	599	603	601	574	667	745–756	541	546
Mg	mmol kg^−1^	14.7	31.4	27.3	26.3	29.5	22.7	40.6	0	0	0–1.3	52.8
Ca	mmol kg^−1^	15	13.4	12.9	13.1	13.2	13.4	11.6	16.4	66.6	26.6–27.4	10.3
Na	mmol kg^−1^	555	513	531	534	536	519	480	589	553	49.4	469
K	mmol kg^−1^	15.5	13.1	14.1	14.1	13.9	13.6	11.4	17.5	20.2–20.4	10.5	10.2
Fe	μmol kg^−1^	18.9	6.6	138	604	392	144	160	N.D.	23,600–25,000	<0.01	0.001
Sr	μmol kg^−1^	92.8	91.0	90.9	90.6	90.1	88.0	86.8	N.D.	200	N.D.	91
Mn	μmol kg^−1^	8	4.6	10.1	12.4	14.2	11.2	9.6	N.D.	2,200–2,350	N.D.	0
Ba	μmol kg^−1^	5.55	2.61	3.23	5.11	5.4	5.43	2.52	8.4	59–79	N.D.	0.07
Li	μmol kg^−1^	175	110	136	140	145	137	69.6	241	327–345	43–46	24.5
Cu	μmol kg^−1^	1.11	0.46	94.5	460	289	4.56	1.39	N.D.	121–162	N.D.	<0.001
H_2_S	mmol kg^−1^	0.927	0.898	0.332	0.439	0.967	0.635	n.d.	N.D.	1.2	0.06	<0.001
SO_4_	mmol kg^−1^	7.85	15.1	12.5	11.7	11.4	10.6	20.1	0	0	0	28.2
^87^Sr/^86^Sr		N.D.	0.706725	0.706174	0.705989	0.705958	0.705873	0.707801	0.702908	N.D.	N.D.	0.7092
K/Cl		0.024	0.022	0.023	0.024	0.023	0.023	0.02	0.026	0.027	0.019	0.019

N.D., not determined; VDVF, Von Damm Vent Field.

VDVF fluid dissolved elemental concentrations from three hydrothermal vents around the active VDVF, with the end-member vent fluid obtained by extrapolation to zero Mg concentration. End-member hydrothermal vent fluid data from the Lost City and Rainbow hydrothermal fields are included for comparison^2^,^3^,^38^,^59^.

**Table 4:** Heat flux calculation.

Mound	Vent orifice	Δ*T* (K)	Radius (m)	Vertical flow rate (m s^−1^)	Calculated flux	Units
Main Cone	Main Hole	87	0.5	1.5	401±80	MW
Main Cone	Spire	211	0.1	1.5	39±10	MW
North spur	Chimlet 2	104	0.1	0.5	6±3	MW
North spur	Chimlet 1	39	0.15	1	11±2	MW
South spur	X-15	108	0.15	1	30±6	MW
Total					487±101	MW

The equation used to calculate the heat flux is *Q*=Δ*T* × *C* × *M*, where: *Q*, heat flux (Watts); Δ*T*, difference in temperature (°C) between the mean temperature of venting hydrothermal fluid and ambient bottom water (4 °C); *C*, specific thermal capacity of seawater at 200 bar and 215 °C (that is, 4,500 J kg^−1 ^K^−1^)^53^; *M*, mass flux (kg s^−1^)=*A × V × ρ*, where *A*, area of vent orifice (m^2^); *V*, mean vertical flow rate (m s^−1^); *ρ*, density of seawater at 215 °C.
