# Supplementary material for: Talc-dominated seafloor deposits reveal a new class of hydrothermal system
Source: Nat Commun. 2015 Dec 22;6:10150. doi: 10.1038/ncomms10150 (PMC4703833; doi:10.1038/ncomms10150)
Supplement: Supplementary Information — Supplementary Figures 1-2 and Supplementary Table 1 [file ncomms10150-s1.pdf]

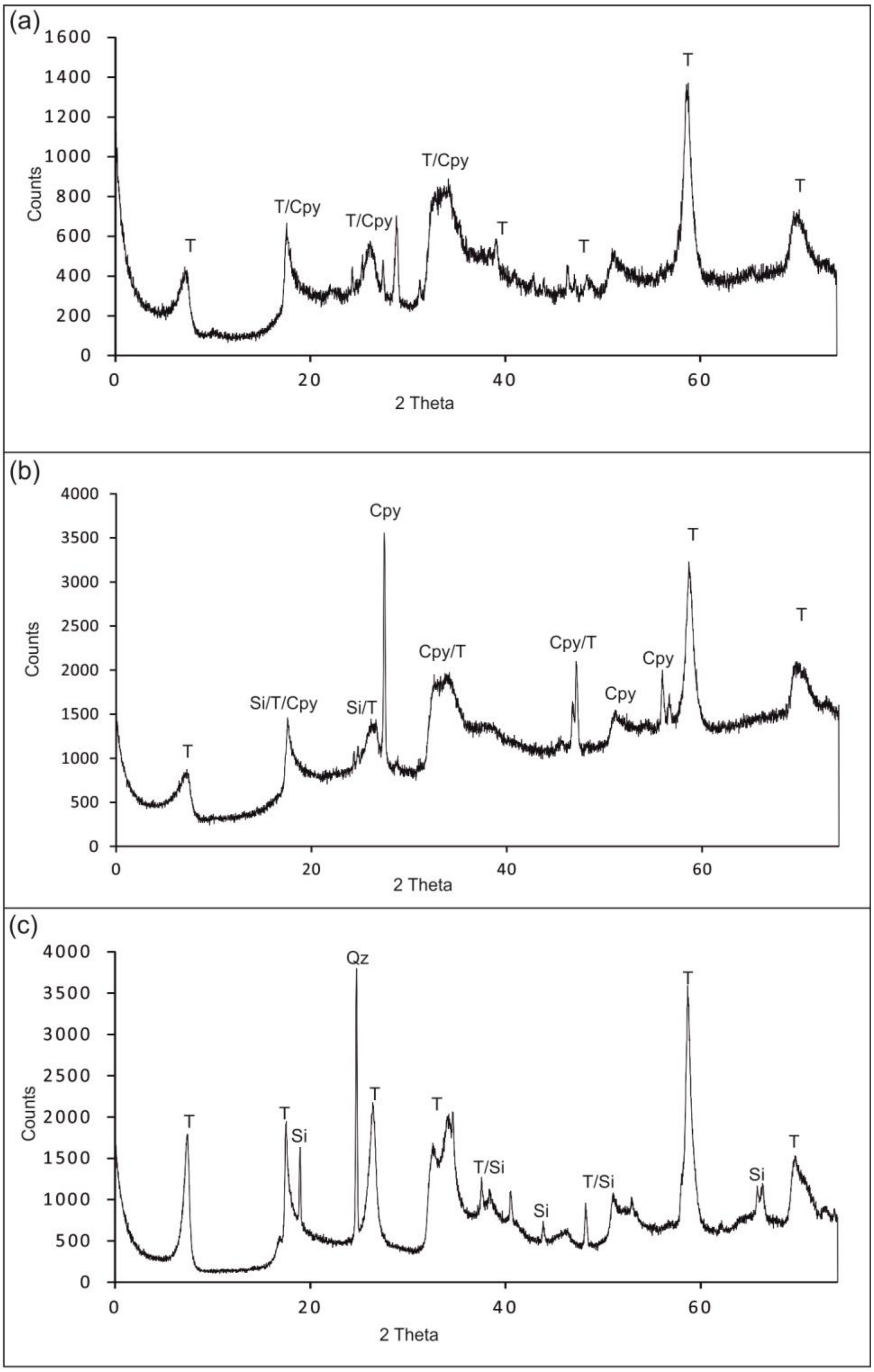

Supplementary Figure 1| Bulk XRD plots. (**a**) Chimney material from the main hole at the base of The Spire, which was venting fluids at 92°C. (**b**) Chimney material from the top of The Spire, which was venting fluids at 215°C. (**c**) Mound breccia material from the rubble apron surrounding the base of the main VDVF mound. Legend: T = talc, Si=microcrystalline silica, Cpy= chalcopyrite

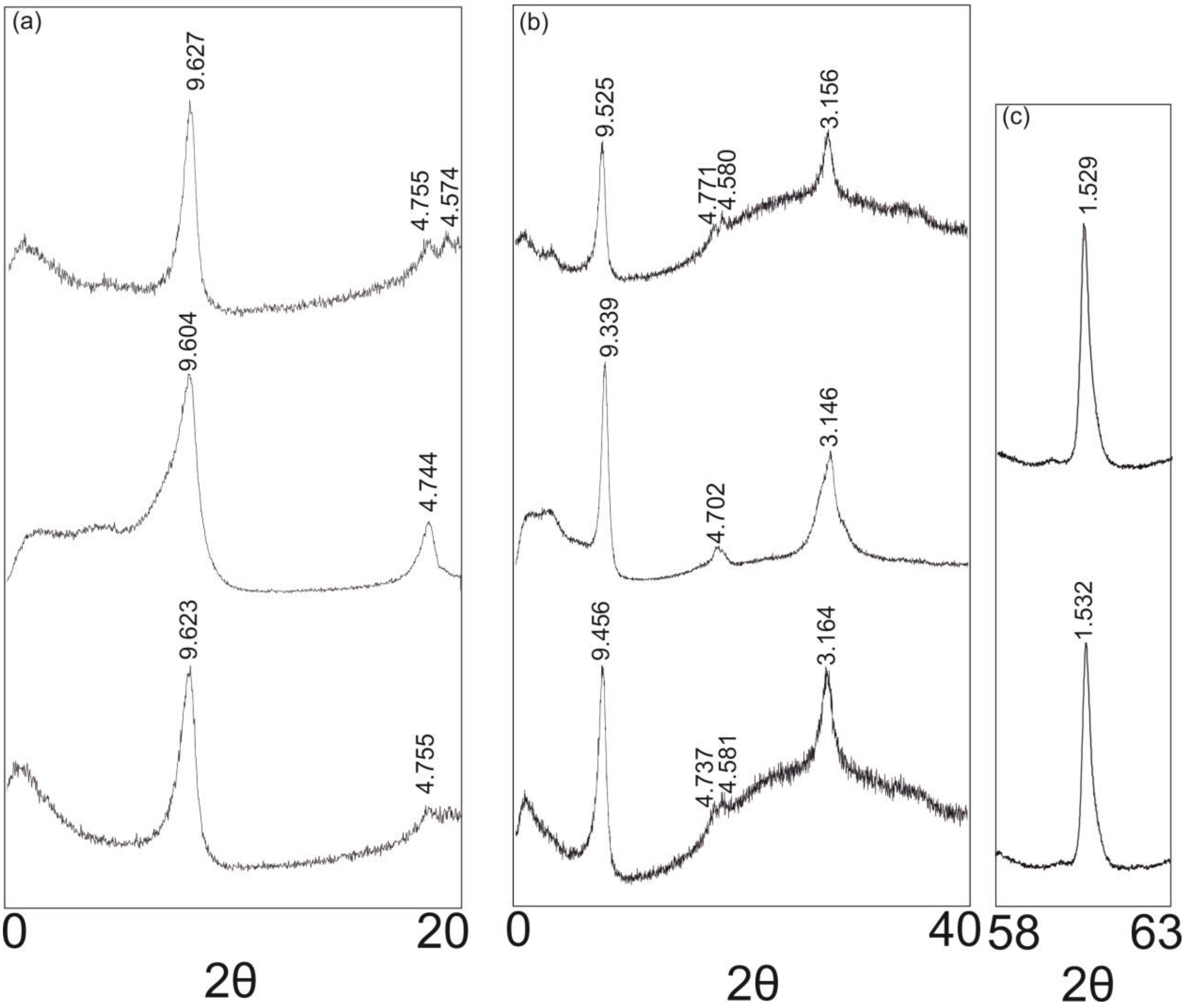

Supplementary Figure 2| Clay XRD plots. (**a**) Orientated air-dried mounts of talc separates. (**b**) Orientated glycolated mounts of talc separates. (**c**) Randomly orientated mounts of talc separates. Glycolated mounts indicate a shift in the (001) peak suggesting the presence of intracrystalline layers of a smectite-group clay. Peaks at ~9.6 Å (001) and ~4.7Å indicate the main phyllosilicate mineral to be talc – the relatively narrow peaks (particularly for (001) peak) suggest that kerolite is absent. (060) peak analysis of randomly orientated mounts indicate a d-spacing of 1.529Å and 1.532Å which is close to the talc peak of 1.527Å but also falls in the range of the tri-octahedral structure (saponite) d-spacing of 1.52-1.54Å and is outside of the di-octahedral (montmorillonite and beidellite) d-spacing range of 1.49-1.50Å.

	Main Hole	Spire	North Spur	North Spur	South Spur
Velocity (m/s)	91°C	215°C	108°C	43°C	112°C
0.3			1		
0.4					
0.5			4		
0.6					
0.7			2		
0.8					2
0.9				4	
1.0					2
1.1		2		2	3
1.2	1			2	
1.3	2				
1.4	2	3			
1.5	4	4			
1.6	2	2			
1.7	2	3			
Average (m/s)	1.5	1.5	0.5	1	1

Supplementary Table 1| Distribution of flow velocities. All recorded measurements of flow velocities from particles in each vent at the VDVF. The averages are reported in Table 4 in the manuscript
